# Whole-genome sequences of *Pseudomonas aeruginosa* strains isolated from patients and inanimate hospital reservoirs in Chattogram, Bangladesh

**DOI:** 10.1128/mra.01517-25

**Published:** 2026-09-09

**Authors:** Sumaiya Hafiz Riana, Kallyan Chakma, Afroza Akter Tanni, Susmita Barua, Sadman Yousuf Sajid, Nahid Sultana, Md. Mustafizur Rahman, Mohammad Jubair, S. M. Rafiqul Islam, Md. Mahbub Hasan, Adnan Mannan

**Affiliations:** 1 Department of Genetic Engineering and Biotechnology, University of Chittagong 54493, Chattogram, Bangladesh; 2 Next-generation Sequencing, Research and Innovation Laboratory Chittagong (NRICh), Disease Biology and Molecular Epidemiology (dBme) Research Group, University of Chittagong 54493, Chattogram, Bangladesh; 3 Department of Microbiology, Chattogram Maa-O-Shishu Hospital, Chattogram, Bangladesh; 4 International Centre for Diarrheal Disease Research, Bangladesh (icddr, b), Dhaka, Bangladesh; Loyola University Chicago, Chicago, Illinois, USA

**Keywords:** *Pseudomonas aeruginosa*, multidrug resistance, Chattogram, Healthcare-associated infections

## Abstract

Antimicrobial-resistant *Pseudomonas aeruginosa* (PA) is a critical concern for the global population. This study identified four isolates and reported the draft genomes annotated as ST645, ST2238, and the high-risk clone ST773. The isolates featured diverse antimicrobial resistance (AMR) gene profiles that emphasize the need for continuous molecular surveillance in Bangladesh.

## ANNOUNCEMENT

*Pseudomonas aeruginosa* (PA) is an opportunistic pathogen from the *Pseudomonadaceae* family and a high critical priority species according to the World Health Organization (1, 2). This report annotates draft genomes of four multidrug-resistant PA isolates collected from a hospital in Chattogram, Bangladesh.

Two hundred thirty-one non-duplicate clinical samples and 97 environmental swab samples were collected from Chattogram Maa-O-Shishu Hospital Medical College (CMOSHMC) between August 2023 and September 2024 (Ethical approval reference CMOSHMC/IRB/2023/06). Clinical samples were obtained by storing pure single colonies of suspected *Pseudomonas* spp. cultured at CMOSH lab in 10% skim milk solution. These stocks were used for the primary culture via the streaking method on *Pseudomonas* Cetrimide Agar (PCA) medium. Environmental swab samples were collected from water taps, basin surfaces, drains, and other environmental sites, and inoculated for primary culture, like the clinical samples. *Pseudomonas* spp. isolates were identified by observing colony morphology and biochemical tests, that is, oxidase, catalase, triple sugar indole test, and citrate test (3). All stocks from cultures were preserved at −20°C. A total of 185 isolates showed morphological and biochemical profiles consistent with PA. Single colonies were selected for subsequent subcultures on PCA medium. The incubation parameters were 37°C for 18–20 h in a conventional incubator. A total of 161 isolates were confirmed as PA via 16S rRNA primer-based conventional PCR (4). Four multidrug-resistant isolates underwent whole-genome sequencing (WGS) based on their antibiogram profiles obtained by performing the Kirby-Bauer disk diffusion method as per the Clinical and Laboratory Standards Institute (CLSI) recommendations (5).

Genomic DNA was extracted from pure colonies of the third subculture PCA medium Petri plates by following the instructions by Qiagen DNeasy Blood & Tissue Kit (Qiagen, #69504). Purity and concentrations were screened by Nanodrop (Thermo Fisher Scientific, ND-1000) spectrophotometer (6). The pair-end library was prepared by Illumina DNA Prep Reagent Kit (version 20060059) via the handler epMotion 5075 as per the manufacturer’s guidelines (7). Qubit 4.0 fluorometer was used for library quantification, and the pooled library was diluted to 4 nM according to the NextSeq System Denature and Dilute Libraries Guide (Illumina Document #15048776, San Diego, CA, USA) (8). Finally, Qubit 4.0 fluorometer was used for quantification, and the NextSeq500 sequencing platform utilizing paired-end 2 × 150 bp reads was used for sequencing.

Raw sequences were run through FastQC v0.12.1 for primary quality assessment, screened and trimmed by FastP v0.23.2, and *de novo* assembly was performed by SPAdes v3.14.1 (9–11). Sequence quality and statistics were obtained via Quast v5.2.0. Prokka v1.13.4 and the Prokaryotic Genome Annotation Pipeline (PGAP) v6.8 were utilized to annotate the assembly (12, 13). Kraken version 1.1.1 confirmed the taxonomic classification (14). Resistance Gene Identifier (RGI) v6.0.3 was used for antibiotic resistance genes (15). All software tools were used under their default parameters.

Total raw read numbers for PA-CU-1, PA-CU-2, PA-CU-3, and PA-CU-5 were 2,294,497, 2,768,576, 3,119,600, and 3,811,318. Sequence analysis by *Pseudomonas aeruginosa* serotyper (PAst) v1.0 revealed that they are sequence types ST645, ST773, ST2238, and ST2238, respectively (16). The genomic characteristics of the *P. aeruginosa* isolates are presented in Table 1. The presence of globally identified high-risk clone ST773 in Bangladesh emphasizes the necessity of further surveillance of PA (17).

**TABLE 1:** Sources, antimicrobial resistance profiles, and genomic data of *Pseudomonas aeruginosa^a^*

Features	PA-CU-1	PA-CU-2	PA-CU-3	PA-CU-5
Sample source				
Source	Patient	Patient	Patient	Hospital reservoir
Specimen	Wound swab	Blood	Blood	Swab of ward basin pipe
Resistance profile				
Isolates resistant to	Fosfomycin, phenicol, fluoroquinolone, sulfonamide, tetracycline, beta-lactam	Aminoglycoside, beta-lactam, fosfomycin, phenicol, fluoroquinolone, cephalosporin	Aminoglycoside, beta-lactam, fosfomycin, phenicol, tetracycline	Beta-lactam, fosfomycin, chloramphenicol, cephalosporin, kanamycin, fluoroquinolone
AMR genes	*fosA, floR, qnrVCq, sul1, tet(G), blaNDM-1, blaOXA-395, blaPAO, floR, qnrVC1, mexD2*	*aph(3′)-Iib, PA emrE, blaOXA-488, blaPAO, fosA, catB7, mexD2, blaPDC-374, crpP*	aph(3′)-llb, blaOXA-396, blaPAO, blaPDC-374, catB7	*blaOXA-485, fosA-3548, catB7, blaPDC-374, aph(3′)-Iib, crpP*
Genome statistics				
Total length	6,780,233	6,447,972	6,314,820	6,604,075
Genome coverage	90.37×	115.22×	132.05×	154.17×
Guanine-Cytosine (%) (GC%)	66	66.5	66.5	66.5
Total contig	229	138	163	133
*N*_50_ values (Kb)	85.2	110.2	87.5	104.2
Genome annotation data				
Genes (total)	6,456	6,103	5,919	6,207
CDSs (total)	6,398	5,996	5,865	6,150
Genes (coding)	6,398	5,996	5,815	6,078
Genes (RNA)	58	58	54	57
Complete rRNAs	1, 1 (5S, 23S)	1, 1 (5S, 16S)	1, 1, 1 (5S, 16S, 23S)	1, 1 (5S, 16S)
tRNAs	50	50	47	49
ncRNAs	4	4	4	4
Pseudogenes (total)	81	49	50	72

^*a*^ CDS, coding sequences; ncRNA, non-coding RNA; PA, *Pseudomonas aeruginosa*.

## Data Availability

The whole-genome analysis data of four PA isolates were deposited in the GenBank repository under BioProject accession number PRJNA1167871. The WGS accession numbers of the reported four assemblies PA-CU-1, PA-CU-2, PA-CU-3, and PA-CU-5 are JBHZPW000000000.1, JBHZPV000000000.1, JBHZPU000000000.1, and JBHZPT000000000.1. The sequences of the isolates are available under SRA accession numbers SRR30858519, SRR30858518, SRR30858517, SRR30858516.
